# Sea Bass Essence from *Lates calcarifer* Improves Exercise Performance and Anti-Fatigue in Mice

**DOI:** 10.3390/metabo12060531

**Published:** 2022-06-08

**Authors:** Hong-Jun Tai, Mon-Chien Lee, Yi-Ju Hsu, Chun-Yen Kuo, Chi-Chang Huang, Ming-Fu Wang

**Affiliations:** 1 Department of Food and Nutrition, Providence University, Taichung 43301, Taiwan; yeast2136@gmail.com; 2 Graduate Institute of Sports Science, National Taiwan Sport University, Taoyuan City 33301, Taiwan; 1061304@ntsu.edu.tw (M.-C.L.); ruby780202@ntsu.edu.tw (Y.-J.H.); 3 Program in Health and Social Welfare for Indigenous Peoples, Providence University, Taichung 43301, Taiwan; cykuo2@pu.edu.tw

**Keywords:** fish, essence, exercise, anti-fatigue

## Abstract

Sea bass (*Lates calcarifer*) is rich in protein, amino acids, and long-chain omega 3 (omega-3), which have many health benefits. In East Asian food culture, soup is often eaten as a nutritional supplement. The purpose of this study was to investigate the benefits of Hi-Q sea bass essence (SBE) supplementation for improved exercise performance and anti-fatigue. Fifty male Institute of Cancer Research (ICR) mice were divided to five groups (10 mice/group) and administered different doses of SBE (EC): (1) vehicle (water); (2) isocaloric (0.94 g casein/kg/mice/day); (3) SBE-1X (1.04 g/kg/mice/day); (4) SBE-2X (2.08 g/kg/mice/day); and (5) SBE-4X (4.16 g/kg/mice/day). We found that SBE supplementation significantly improved more than 1.96-fold endurance exercise performance (*p* < 0.05) and more than 1.13-fold glycogen storage in the liver and muscles (*p* < 0.05), and had dose-dependent by SBE dose (*p* < 0.05). In addition, supplementation with SBE at different doses had significant effects on the fatigue-related biochemical markers, i.e., lactate, ammonia, and blood urea nitrogen (BUN) levels were reduced significantly (*p* < 0.05), and were also dose-dependent. In conclusion, supplementation with SBE for 4 weeks was able to effectively improve exercise performance and had an anti-fatigue effect. In addition, it did not cause any physiological or histopathological damage.

## 1. Introduction

Fatigue is a common and complex non-specific physiological phenomenon defined as the inability to maintain power output and strength, and includes central nervous system fatigue and peripheral fatigue, which can lead to serious health problems [1]. During exercise, with the prolongation of exercise time or an increase in exercise intensity, stored energy reserves are rapidly depleted. This can lead to a shift from aerobic metabolism to anaerobic metabolism [2]. At this point, glycogen in the liver and muscles is metabolized to glucose by the lactic acid energy system, which is further metabolized to meet the higher energy demands, and in this state, the body produces large amounts of lactate [3]. Muscle fatigue occurs when metabolites such as lactate, ammonia, blood urea nitrogen (BUN), and inorganic phosphorus accumulate to cause intracellular acidosis, and imbalances in reactive oxygen species (ROS) levels, internal pH, and osmotic pressure [4]. At this time, the body cannot maintain the energy supply, and a large amount of fatigue metabolites accumulate, which leads to a decline in exercise performance [5]. To avoid this problem, regular exercise training combined with a balanced diet can help delay and prevent fatigue during exercise [6], and previous research has demonstrated that nutritional supplements developed from natural food extracts can improve athletic performance, reduce fatigue, and speed up recovery [7].

The intake of nutrients is not only necessary for growth and the maintenance of life, but is also closely related to physical fatigue and energy metabolism [8]. Previous studies found that protein, amino acid, and active peptide supplementation can reduce the accumulation of harmful metabolites, increase antioxidant levels, and reduce fatigue [9,10]. Among them, fish meat is rich in nutrients and is a source of high-quality protein, minerals, and essential fatty acids, especially unsaturated fatty acids, such as docosahexaenoic acid (DHA, C22:6n3) and eicosapentaenoic acid (EPA, C20:5n3) [11]. In addition, fish meat protein is more digestible than the majority of terrestrial meat proteins and is richer in essential amino acids [12]. Previously study has shown that fish protein hydrolysate (FPH) has antioxidant properties, mainly resulting from dipeptides and tripeptides, which are more readily absorbed than free amino acids and intact proteins [13], and also has anti-hypertension, anti-cancer, anti-inflammatory, and anti-bacterial properties and other effects [14]. As compared with whey protein hydrolysate of equal weight, FPH has a higher total antioxidant capacity [15]. However, fish meat is extremely difficult to preserve and is easily spoiled after being caught. Therefore, different processing methods are needed to improve its shelf life [16].

Boiling into soup is one of the important ways to preserve food, and it can also preserve the nutritional content of food. In East Asia, soup is one of the most important components of the food culture. It has different properties and nutrient contents after cooking, depending on the items used, and plays a vital role in the health and maintenance of the body. In addition, drinking soup can increase satiety, help people stay fit, and reduce the incidence of obesity [17]. In addition, bioactive compounds and peptides released during digestion, thermal pretreatment, microbial fermentation, and other technological processing further enhance the bioactivity of foods [18]. In particular, hot-processed ready-to-drink gravies or flavored soups are currently very popular around the world. Processing at high temperatures and pressures for long periods (≥5 h) helps to decompose macro-molecules in meat into micro- or nano-sized particles, effectively inhibiting free radicals in the body and thus reducing the incidence of related conditions [19].

Asia sea bass (*Lates calcarifer*) is an economically important fish in Southeast Asia and it contains high levels of protein and essential amino acids [20]. Moreover, it contains many health-promoting polyunsaturated fatty acids, including omega-3 and omega-6 [21]. Simmering sea bass soup for long periods does not affect the free amino acids and essential amino acids, helps to increase the availability of phenolic substances, amino acids, and Maillard reaction products (MRPs), and has antioxidant effects [22]. Currently, it is often used as a nutritional supplement for pregnant women, postpartum women, the elderly, and frail and postoperative patients to enhance energy and physique. However, there are still very few fish-related products used as sports nutrition supplements. Therefore, the purpose of this study was to explore the potential benefits of sea bass extract (SBE) supplementation for 4 weeks in terms of improving exercise performance and anti-fatigue in order to elucidate the underlying mechanisms of the anti-fatigue effects and to assess whether there are adverse effects on the body.

## 2. Results

### 2.1. Effect of SBE Supplementation on Exercise Performance

**Figure 1:**
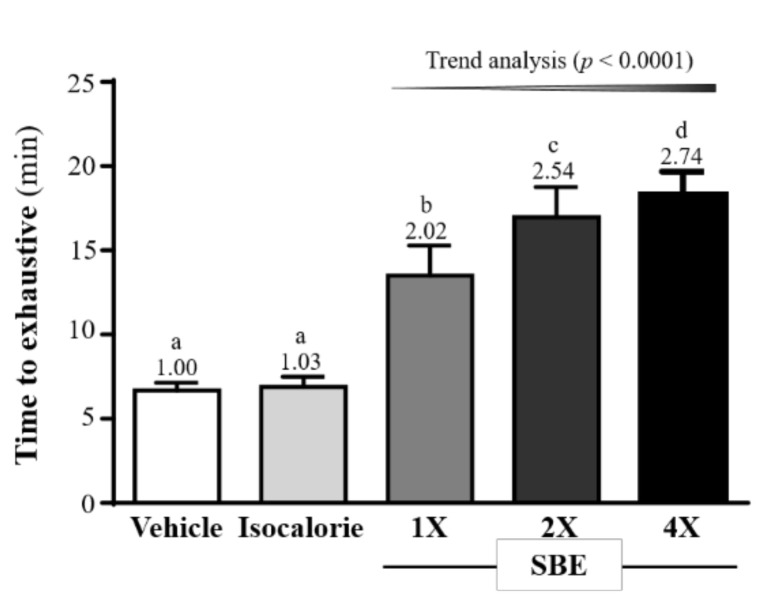
Effect of supplementation with SBE on exhaustive swimming time in mice. Data are expressed as mean ± SD (*n* = 10 mice per group). The different superscript letters (a, b, c, d) above each bar indicate a significant difference between the groups (*p* < 0.05).

As shown on Figure 1, the exhaustive swimming times for the vehicle, isocaloric, SBE-1X, SBE-2X, and SBE-4X groups were 6.68 ± 0.46, 6.89 ± 0.59, 13.48 ± 1.80, 16.97 ± 1.78, and 18.33 ± 1.34 min, respectively. The values from the SBE-1X, SBE-2X, and SBE-4X groups were 2.02-fold, 2.54-fold, and 2.74-fold (*p* < 0.0001) higher, respectively, than those of the vehicle group. In addition, they were 1.96-fold, 2.46-fold, and 2.66-fold higher (*p* < 0.0001), respectively, than the values from the isocaloric group. For the trend analysis, exhaustive swimming time dose-dependently increased with SBE supplementation (*p* < 0.0001).

### 2.2. Effect of SBE Supplementation on Serum Lactate Levels after the 10-min Swim Test

Before swimming, the serum lactate level differences between each group were not significant (*p* > 0.05). After 10 min of swimming, the serum lactate levels in the SBE-1X, SBE-2X, and SBE-4X groups were 18.90% (*p* = 0.0007), 23.60% (*p* < 0.0001), and 29.00% (*p* < 0.0001) lower, respectively, than those in the vehicle group. In addition, they were lower than the isocaloric group by 18.30% (*p* = 0.0009), 23.00% (*p* < 0.0001), and 28.40% (*p* < 0.0001), respectively. The lactate production rate was calculated from the lactate levels before and 10 min after exercise. The results suggest that, as compared with vehicle group, the SBE-1X, SBE-2X, and SBE-4X groups were significantly decreased by 16.20% (*p* = 0.0046), 22.50% (*p* = 0.0002), and 29.50% (*p* = 0.0001), respectively. Moreover, the values from the SBE-1X, SBE-2X, and SBE-4X groups were 18.10% (*p* = 0.0015), 24.30% (*p* < 0.0001), and 31.10% (*p* < 0.0001) lower, respectively, than those of the isocaloric group. At 10 min after swimming, the lactate production rate had decreased dose-dependently with SBE supplementation, with a significant trend (*p* < 0.0001) (Table 1).

After 20 min of resting following the swimming test, the serum lactate levels in the SBE-1X, SBE-2X, and SBE-4X groups were significantly decreased as compared with the vehicle group, i.e., by 16.30% (*p* = 0.0006), 25.90% (*p* < 0.0001), and 34.50% (*p* < 0.0001), respectively. They were also 17.00% (*p* = 0.0009), 26.50% (*p* < 0.0001), and 35.00% (*p* < 0.0001) lower, respectively, than those of the isocaloric group, and had decreased dose-dependently with SBE supplementation, with a significant trend (*p* < 0.0001). However, the clearance rates (the recovery effect of lactate after 10 min of exercise followed by 20 min of rest) in the vehicle, isocaloric, SBE-1X, SBE-2X, and SBE-4X groups were 0.19 ± 0.12, 0.18 ± 0.13, 0.16 ± 0.16, 0.23 ± 0.09, and 0.26 ± 0.08 (mmol/L). There was no significant difference between groups (Table 1).

### 2.3. Effect of SBE Supplementation on Fatigue-Related Biochemical Indicators after the 10-min Swim Test or a 90-min Swim Test and a 60-min Rest

**Figure 2:**
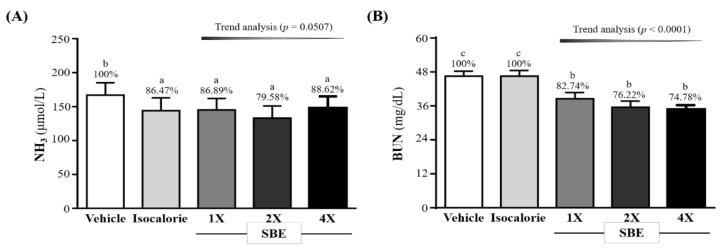
Effect of supplementation with SBE on serum (**A**) NH_3_ and (**B**) BUN. Data are expressed as mean ± SD for *n* = 10 mice per group. The different superscript letters (a, b, c) above each bar indicate a significant difference at *p* < 0.05. NH_3_: blood ammonia; BUN: blood urea nitrogen.

We also evaluated the NH_3_ and BUN concentration after the 10-min swim test. As shown in Figure 2A, the NH_3_ levels in the vehicle, isocaloric, SBE-1X, SBE-2X, and SBE-4X groups were 167 ± 18, 144 ± 19, 145 ± 17, 133 ± 18, and 148 ± 17 (umol/L), respectively. The SBE-1X, SBE-2X, and SBE-4X groups were significantly lower than the vehicle group, i.e., by 13.11% (*p* = 0.0089), 20.42% (*p* = 0.0001), and 11.38% (*p* = 0.0221), respectively, but no dose-dependent trend was observed.

We measured the serum BUN level after the 90-min swim test followed by 60 min of rest. As shown in Figure 2B, the BUN levels in the SBE-1X, SBE-2X, and SBE-4X groups were 46.5 ± 1.8, 46.5 ± 2.0, 38.5 ± 2.2, 35.5 ± 2.2, and 34.8 ± 1.5 (mg/dL), respectively. Compared with the vehicle group, the SBE-1X, SBE-2X, and SBE-4X groups were significantly lower by 17.26% (*p* < 0.0001), 23.78% (*p* < 0.0001), and 25.22% (*p* < 0.0001), respectively. In addition, they were significantly lower than the isocaloric group by 17.27% (*p* < 0.0001), 23.79% (*p* < 0.0001), and 25.23% (*p* < 0.0001), respectively. For the trend analysis, serum BUN levels after the 90-min swim test followed by 60 min of rest had decreased dose-dependently with SBE supplementation (*p* < 0.0001).

### 2.4. Effect of SBE Supplementation on Liver and Muscle Glycogen Contents

**Figure 3:**
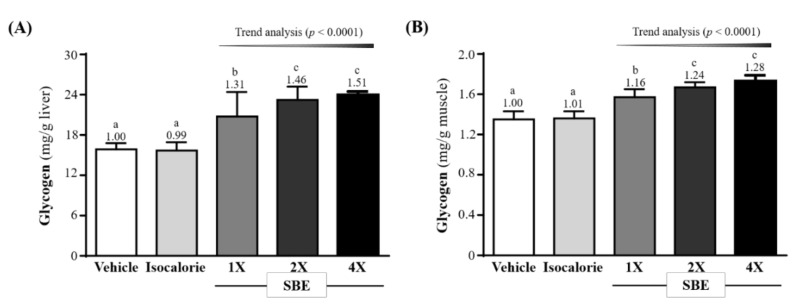
Effect of supplementation with SBE on (**A**) liver and (**B**) muscle glycogen. Data are expressed as mean ± SD for *n* = 10 mice per group. The different superscript letters (a, b, c) above each bar indicate a significant difference at *p* < 0.05.

The liver glycogen content in the vehicle, isocaloric, SBE-1X, SBE-2X, and SBE-4X groups were 15.87 ± 0.93, 15.69 ± 1.24, 20.76 ± 3.65, 23.24 ± 1.98, and 23.97 ± 0.51 (mg/g liver), respectively. The SBE-1X, SBE-2X, and SBE-4X groups were significantly greater than the vehicle group by 1.31-fold (*p* < 0.0001), 1.46-fold (*p* < 0.0001) and 1.51-fold (*p* < 0.0001), respectively, and also were significantly greater than the isocaloric group by 1.32-fold (*p* < 0.0001), 1.48-fold (*p* < 0.0001) and 1.53-fold (*p* < 0.0001), respectively (Figure 3A).

The muscle glycogen content in the vehicle, isocaloric, SBE-1X, SBE-2X, and SBE-4X groups were 1.35 ± 0.08, 1.36 ± 0.07, 1.57 ± 0.08, 1.67 ± 0.05, and 1.73 ± 0.06 (mg/g muscle), respectively. The SBE-1X, SBE-2X, and SBE-4X groups were significantly greater than the vehicle group by 1.16-fold (*p* < 0.0001), 1.24-fold (*p* < 0.0001) and 1.28-fold (*p* < 0.0001), respectively, and were also significantly greater than the isocaloric group by 1.15-fold (*p* < 0.0001), 1.22-fold (*p* < 0.0001) and 1.26-fold (*p* < 0.0001), respectively (Figure 3A).

In the trend analysis, SBE supplementation dose-dependently increased liver and muscle glycogen contents (*p* < 0.0001).

### 2.5. Effect of SBE Supplementation on Biochemical Variables at the End of the Experiment

We assessed whether 4 weeks of SBE supplementation caused biochemical changes in the blood. The results showed that there was no significant difference between the groups in terms of liver function, renal function, blood lipids, and other indicators (*p* > 0.05) (Table 2).

### 2.6. Subchronic Toxicity Evaluation of SBE Supplementation

**Figure 4:**
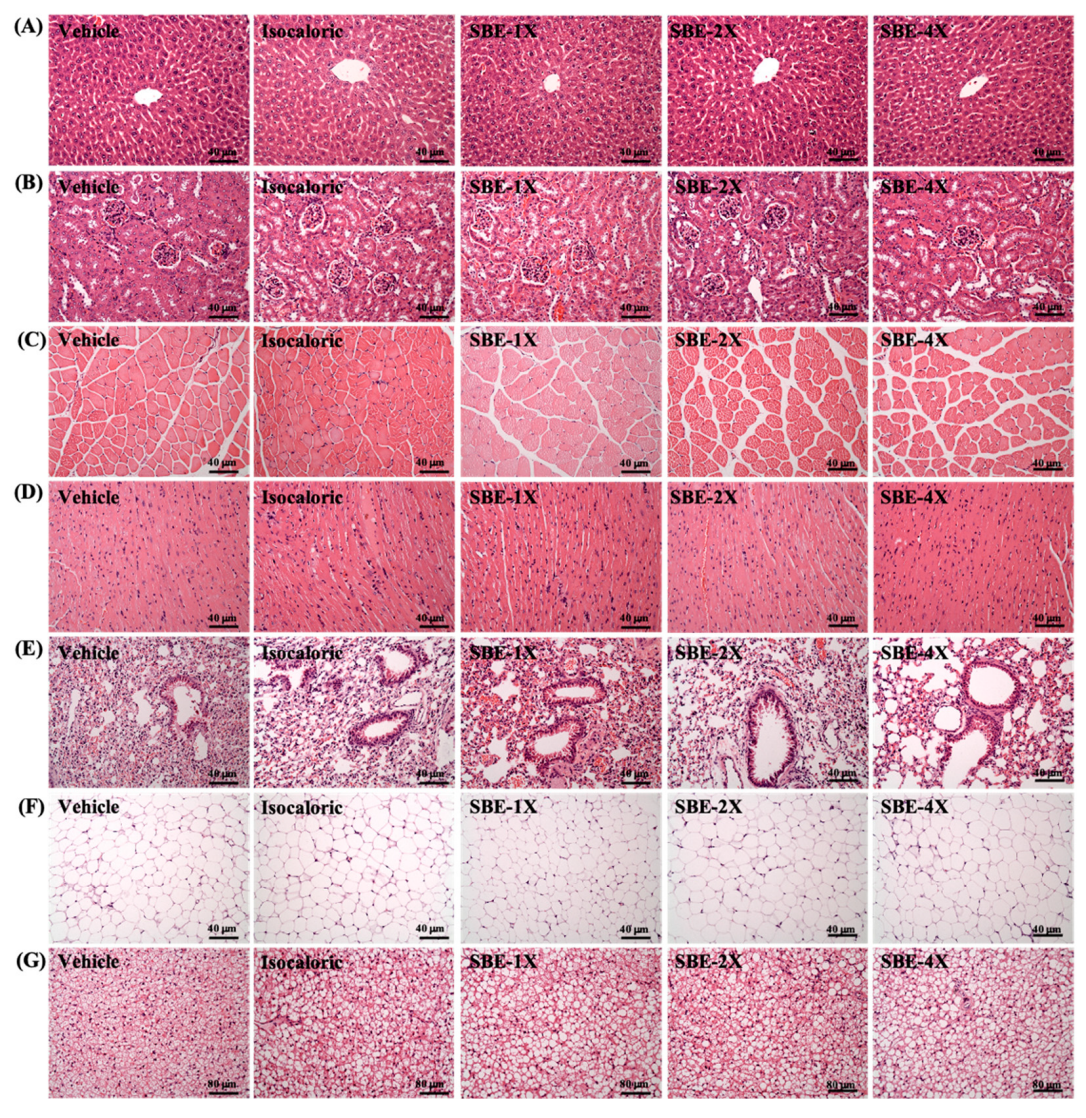
Effect of SBE supplementation on (**A**) liver, (**B**) kidney, (**C**) muscle, (**D**) heart, (**E**) lung, (**F**) adipocyte tissue, and (**G**) BAT tissue in mice. H&E stain, magnification: 200×; bar, 40 μm; BAT magnification: 100×; bar, 80 μm.

As shown on Table 3, after supplementation with SBE for 4 weeks, the weight of mice in each group exhibited a steady increase every week. Among them, there was no significant difference in mouse water intake in each group in the 4 weeks after SBE intervention (*p* > 0.05). There was also no significant difference in tissue weight among mice, which exhibited a relatively steady weight gain. Therefore, SBE supplementation for 4 consecutive weeks did not cause any organ hypertrophy or atrophy. In addition, as can be observed from the histopathological section results in Figure 4, the livers, kidneys, muscles, hearts, lungs, EFP, and BAT of the mice did not exhibit abnormalities in any group. Therefore, we confirmed that SBE had no adverse effects on organs and tissues at the doses tested in this study.

## 3. Discussion

At present, the majority of studies on the anti-fatigue effects of meat protein sources focus on terrestrial animals [23]. Moreover, studies on chicken essence account for the vast majority of meat essence-related research [24]. A previous study noted that, despite the same efficacy, various bioactive peptides differ between meat sources and that different boiling processes have an effect on nutritional content [25]. Although there are currently few reports on the anti-fatigue properties of fish-related products, in this study, we demonstrated that 4 consecutive weeks of SBE supplementation significantly improved the exercise performance, glycogen storage, and significantly reduced post-exercise fatigue metabolite production and accumulation in mice. In addition, we confirmed that SBE supplementation does not adversely affect the organs or tissues of mice.

Past research demonstrated that fish protein is easy to digest and rich in animal-derived protein, essential amino acids, and the long-chain omega-3s found in polyunsaturated fatty acids (PUFAs) [26]. The SBE supplements in this study were rich in branched-chain amino acid (BCAA), which are considered to be important for tissue synthesis, energy supply, and health maintenance [27]. Previous studies noted that leucine and isoleucine can be metabolized to acetoacetyl-CoA through transamination (TA) and enter the citric acid cycle to generate more energy for working muscles [28]. In addition, isoleucine and valine can be converted into α-keto acid by transamination, metabolized to succinyl-CoA, converted into malate and pyruvate, and finally converted into alanine [29]. Alanine is a dispensable amino acid that is synthesized endogenously by the liver and acts as an auxiliary energy source in extreme situations, such as starvation and prolonged endurance exercise [30]. Alanine is shuttled through the blood to the liver, converted into pyruvate through a transamination reaction, and catalyzed by glutamate–pyruvate transaminase [31]. Pyruvate can then serve as a metabolic substrate through the gluconeogenesis pathway, where newly formed glucose can promote muscle formation. This conversion pathway is known as the glucose–alanine cycle [32], and although BCAA is not as direct as sugar supplementation, in terms of increasing hepatic glucose storage, it has a positive effect. A previous study showed that 6 consecutive weeks of BCAA-enriched supplementation with exercise training significantly increased hepatic glycogen storage in rats [33]. This is in accordance with the results of this study that suggest that CAA-enriched SBE has the effect of significantly increasing glycogen storage in the liver and muscles of mice after 4 consecutive weeks of supplementation (Figure 3).

During prolonged or vigorous exercise, large amounts of ATP are depleted, and muscle contractions activate AMP-activated protein kinase (AMPK) by increasing the cellular AMP/ATP ratio [34]. Activation of AMPK inhibits the ATP utilization pathways and promotes the ATP-producing pathways, which are critical for endurance exercise [35]. Therefore, during high-intensity exercise, which is highly dependent on glycogenolysis, glycogen availability is critical to facilitate ATP resynthesis. Glycogen is considered the primary fuel source during prolonged moderate- and high-intensity endurance exercise [36]. When glucose levels are low, the glycogen stored in the liver and muscles replenishes the glucose needed by the body through the glycolytic pathway. Therefore, the more glycogen stored in the body, the more glucose available to maintain blood circulation, and the better the exercise performance [37]. According to research, BCAAs may play a role in glycogen metabolism during prolonged exercise, as supplementation of these amino acids preserves liver and muscle glycogen, thereby improving exercise performance [38]. In previous human trials, 7-day BCAA-containing beverage supplementation significantly increased VO_2max_ and power output [39]. In another study, BCAA-enriched chicken essence supplementation in mice for 4 weeks not only significantly increased glycogen stores, but also significantly improved exercise endurance performance. Additionally, it has the benefit of improving biochemical markers of post-exercise fatigue [40]. Similar to our findings, in addition to a significant increase in glycogen, SBE significantly improved exercise endurance performance after 4 consecutive weeks of supplementation (Figure 1). Better exercise performance can delay fatigue, and delaying fatigue can improve exercise performance. Results from a previous study found that BCAA supplementation combined with swimming training for 6 weeks promoted a significant increase in liver and muscle glycogen storage and significantly prolonged exercise-to-failure time as compared to a sedentary control group [41]. Additionally, BCAA supplementation may delay CNS fatigue and improve aerobic endurance performance by increasing the ratio of free tryptophan and reducing serotonin synthesis in the brain [42].

In past studies, lactate, ammonia, and BUN levels increasing with exercise duration and intensity and recovering at rest were often used as indicators of post-exercise muscle fatigue [43]. Among them, lactate is the result of anaerobic metabolism of glucose during exercise and is one of the important indicators with which to judge muscle fatigue and muscle activity limiting factors [44]. During prolonged or strenuous exercise, the H^+^ concentration increases and the pH in blood and muscle tissue decreases, thereby inhibiting glycolysis. In addition, Ca^2+^ release is associated with muscle contraction, causing various metabolic and physiological side effects, leading to muscle damage and decreased exercise capacity [45]. Furthermore, during high-intensity exercise, muscles must obtain sufficient energy from anaerobic glycolysis, which produces lactate from glycolytic metabolism. Lactic acid is an oxidizable substrate in the skeletal muscle and a precursor to gluconeogenesis in muscles or the liver after exercise. As exercise progresses, the amount of oxygen absorbed and delivered by muscle tissue decreases, thereby regulating the body by preventing pyruvate from efficiently entering the TCA cycle and converting it into lactate. In previous studies, BCAA supplementation has been shown to significantly reduce post-exercise blood lactate concentrations [44]. A study in athletes took BCAA at 0.2 g/kg BW for one month found significantly lower blood lactate concentrations after exercise compared to placebo group [46]. Another study found that BCAA supplementation significantly reduced blood lactate concentrations after prolonged exercise compared to control group [47]. This appears to confirm that, in the current study, 4 consecutive weeks of SBE supplementation significantly reduced post-exercise lactate concentrations and decreased the lactate product rate (Table 1). Another indication that ammonia is a ubiquitous metabolite after exercise. Adenosine monophosphate (AMP) is converted into inosine monophosphate (IMP) during ATP resynthesis when the availability of adenosine triphosphate (ATP) exceeds the rate of ATP production. At this time, during high-intensity or long-term exercise, ammonia in the skeletal muscle significantly increases and accumulates, mainly due to the increased activity of purine nucleotide cycling in the skeletal muscle [48,49]. Ammonia is metabolized to BUN through the urea cycle, so BUN is not only a marker of renal function, but can also be considered a biomarker of ATP metabolism [50]. The results of this study were validated in previous trials, in which BCAAs were administered with a significant reduction in post-exercise ammonia levels [51]. Another study gave rats 6 weeks of exercise training (5 days/week) combined with a 4.76% BCAA diet and found that had a beneficial effect on performance by sparing glycogen in the soleus muscle (*p* < 0.05) and by inducing a lower concentration of plasma ammonia [41]. In our previous study, after 4 weeks of continuous chicken essence supplementation, it was found that the concentrations of lactate and NH_3_, and the BUN level after exercise were significantly reduced in mice. In addition, it had the effect of improving exercise performance [24]. When we supplemented mice with SBE for 4 consecutive weeks, we observed a similar effect, i.e., significantly reduced post-exercise NH_3_ and BUN concentrations (Figure 2A,B).

In the current study found that after 4 consecutive weeks of SBE supplementation, the analysis of blood parameters confirmed that there were no significant differences in liver function, renal function, and blood lipid-related indexes between different doses of SBE groups, which were all within a reasonable range (Table 2). In addition, no tissue damage, lesions, or fat accumulation were found in the liver, kidney, heart, and other parts through pathological section observation. Therefore, we do not believe that SBE supplementation will cause any adverse harm (Figure 4).

Although in this study we demonstrated that SBE helps improve exercise performance and delay exercise fatigue; however, there is currently little research on meat protein as a nutritional supplement. This is especially true of fish protein and may be related to the high cost of edible farming and aquaculture [52]. Nevertheless, the use of food industry technology to extract fish protein and retain special biologically active peptides is an important field of study. We expect that under the scope of sustainable management, further marine resources can be discovered that help improve sports performance, anti-fatigue supplements, and other efficacy mechanisms, and can also improve the utilization rate of food.

## 4. Materials and Methods

### 4.1. Hi-Q Sea Bass Essence (SBE) Preparation

**Figure 5:**
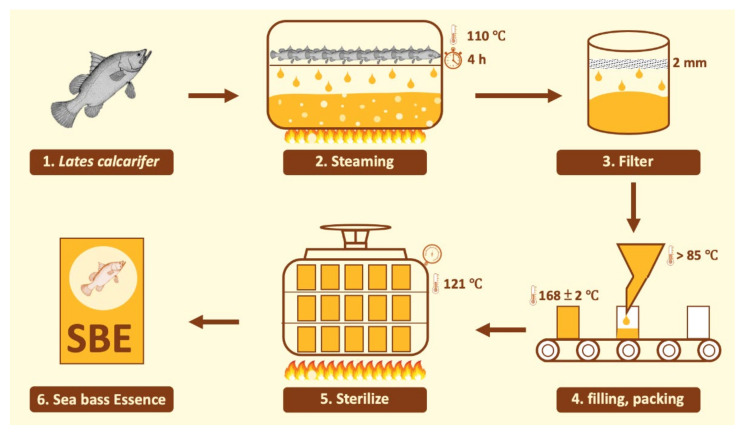
SBE production process.

Hi-Q sea bass essence (SBE), which is processed using a range of food technologies, was provided by Hi-Q Marine Biotech International Ltd. (Taipei, Taiwan). The detailed process is shown in Figure 5. The recommended daily intake of SBE for an adult weighing 60 kg is 60 mL (1 mL/kg body weight). However, in this study, SBE was lyophilized for precise supplementation in animals. After 60 mL of the product was lyophilized, 5.07 g of lyophilized solid was obtained (8.45% freeze-dried rate). The nutritional and total branched-chain amino acids (BCAA) data of SBE were confirmed by SGS Taiwan, Ltd. (New Taipei City, Taiwan) and are shown in Table 4.

In this study, the SBE dose designed for humans was 5.07 g per day (lyophilized powder). However, a conversion factor of 12.3 was used to account for the difference between the body surface area of mice and humans, according to suggestions from the US Food and Drug Administration. After detailed calculations, we concluded that the daily 1X dose for mice was to be 845 mg/kg. We administered 1X, 2X, and 4X doses in this study to compare the benefits of different doses. In addition, we added an isocaloric group to eliminate the effects of supplemental calories.

### 4.2. Experimental Design

We used male institute of cancer research (ICR) mice (6 weeks old, 25–28 g/mouse) from BioLASCO Taiwan (Yi-Lan Breeding Center, Yi-Lan County, Taiwan). All mice were maintained at 12-h light/12-h dark cycle at room temperature (22 ± 2 °C) and 50–60% humidity. They were given a standard laboratory diet (No. 5001; PMI Nutrition International, Brentwood, MO, USA) and distilled water ad libitum, and were allowed food ad libitum for 2 weeks prior to the experiments. The Institutional Animal Care and Use Committee (IACUC) of National Taiwan Sport University approved this experiment (IACUC-10910). In total, 50 mice were randomly assigned to 5 groups (10 mice/group) for oral gavage treatment for 4 weeks: (1) vehicle (vehicle control or water only); (2) isocaloric (0.94 g casein/kg/mice/day); (3) SBE-1X (1.04 g/kg/mice/day); (4) SBE-2X (2.08 g/kg/mice/day); and (5) SBE-4X (4.16 g/kg/mice/day). The body weight, water consumption, and food intake were recorded each week.

### 4.3. Swimming Exercise Performance Test

All mice were loaded with a piece of lead that weighed 5% of the mouse’s body weight (BW) on the tail. They were then individually placed in a cylindrical swimming pool (65 cm high, 20 cm radius) that was filled with water to a depth of 40 cm and maintained at 27 ± 1 °C. We recorded time until mouse exhaustion as the swimming endurance time. Fatigue was defined as loss of coordinated movement or failure to return to the surface within 8 s, as previously described [53].

### 4.4. Determination of Fatigue-Associated Biochemical Variables

The effects of SBE supplementation on fatigue-associated biochemical indices were evaluated pre-exercise, post-exercise, and during rest. As previously described [54], all mice were fasted for 12 h and blood samples were collected to analyze lactate, blood ammonia (NH_3_), and glucose at baseline, after swimming unloaded for 10 min, and after resting for 20 min. In addition, we evaluated blood urine nitrogen (BUN) after 90 min of prolonged exercise and 60 min of rest. The serum was collected by centrifugation at 1500× *g* for 15 min from the blood and was measured with an automatic analyzer (model 7060, Hitachi, Tokyo, Japan).

### 4.5. Clinical Biochemical Profiles

Thirty minutes after the final supplementation, all mice were euthanized using 95% CO_2_ and blood samples were collected immediately. After centrifugation to collect serum, the clinical biochemical variables, including aspartate aminotransferase (AST), alanine transaminase (ALT), albumin, triglycerides (TG), blood urea nitrogen (BUN), creatinine, uric acid (UA), total protein (TP), CK, and glucose, were measured using an autoanalyzer (model 7060, Hitachi, Tokyo, Japan).

### 4.6. Visceral Tissue Weight and Histology Staining and Glycogen Determination

The liver, kidneys, heart, lungs, muscles, epididymal fat pad (EFP), and brown adipose tissue (BAT) of mice were excised and weighed post-euthanization. We carefully removed, chopped, and fixed of all the tissue in 10% formalin, and then embedded it in paraffin and cut it into 4-μm-thick sections for morphological and pathological evaluation. Furthermore, we used hematoxylin and eosin (H&E) to stain the sections and then a veterinary pathologist using an optical microscope equipped with a CCD camera (BX-51, Olympus, Tokyo, Japan) examined them. Parts of the muscle and liver tissues were stored in liquid nitrogen for glycogen content analysis, as previously described [55].

### 4.7. Statistical Analysis

We used the statistical analyses software SAS 9.4 (SAS Inst., Cary, NC, USA) to calculate the statistical differences among groups. One-way analysis of variance (ANOVA) and the Cochran–Armitage test were used for the dose–effect trend analysis. All data are expressed as mean ± SD for *n* = 10 mice per group. *p* < 0.05 was considered statistically significant.

## 5. Conclusions

In conclusion, we found that supplementation with SBE for 4 consecutive weeks not only did not cause any physiological and pathological harm, but significantly improved exercise endurance performance and glycogen storage. SBE could also significantly reduce post-exercise fatigue biochemical markers, such as blood ammonia, lactate, and BUN in a dose-dependent manner. Nevertheless, the use of food industry technology to extract fish protein and retain special biologically active peptides is an important field of study. This study not only confirms the benefits of meat protein as a nutritional supplement for improving exercise performance and anti-fatigue, but also increases the future research and application of meat protein food processing products to further explore the molecular mechanism of its action.

## Figures and Tables

**Table 1:** Effects of SBE supplementation on serum levels of lactate after the 10 min swim test. The lactate production rate (B/A) was the value of the lactate level after exercise (B) divided by that before exercise (A). The clearance rate (B − C)/B was defined as the lactate level after swimming (B) minus that after 20 min of rest (C) divided by that after swimming (B). Data are expressed as mean ± SD (*n* = 10 mice per group). Values in the same row with different superscript letters (a, b, c, d) differ significantly between groups, *p* < 0.05.

	Groups	Vehicle	Isocaloric	SBE-1X	SBE-2X	SBE-4X
Time Point		Lactate (mmol/L)				
Before swimming (A)		3.81 ± 0.42 ^a^	3.69 ± 0.46 ^a^	3.70 ± 0.35 ^a^	3.77 ± 0.49 ^a^	3.86 ± 0.51 ^a^
After swimming (B)		6.56 ± 0.93 ^b^	6.51 ± 1.01 ^b^	5.32 ± 0.89 ^a^	5.01 ± 0.28 ^a^	4.66 ± 0.37 ^a^
After a 20 min resting (C)		5.21 ± 0.53 ^d^	5.25 ± 0.72 ^d^	4.36 ± 0.48 ^c^	3.86 ± 0.46 ^b^	3.41 ± 0.26 ^a^
Rates of lactate production and clearance						
Production rate = B/A		1.73 ± 0.25 ^c^	1.77 ± 0.21 ^c^	1.45 ± 0.28 ^b^	1.34 ± 0.14 ^ab^	1.22 ± 0.11 ^a^
Clearance rate = (B − C)/B		0.19 ± 0.12 ^a^	0.18 ± 0.13 ^a^	0.16 ± 0.16 ^a^	0.23 ± 0.09 ^a^	0.26 ± 0.08 ^a^

**Table 2:** Effects of SBE supplementation on biochemical parameters.

	Groups	Vehicle	Isocaloric	SBE-1X	SBE-2X	SBE-4X
Parameters						
AST (U/L)		73 ± 11	71 ± 7	75 ± 8	72 ± 6	74 ± 5
ALT (U/L)		47 ± 5	47 ± 5	49 ± 5	47 ± 5	47 ± 5
ALB (g/dL)		3.44 ± 0.11	3.28 ± 0.23	3.37 ± 0.24	3.37 ± 0.32	3.35 ± 0.21
BUN (mg/dL)		27.1 ± 3.7	26.1 ± 1.9	26.3 ± 2.3	26.2 ± 2.6	26.3 ± 2.7
CREA (mg/dL)		0.43 ± 0.02	0.43 ± 0.02	0.44 ± 0.03	0.44 ± 0.03	0.43 ± 0.03
UA (mg/dL)		2.1 ± 0.8	2.1 ± 0.5	2.0 ± 0.5	2.2 ± 0.4	2.1 ± 0.8
TP (g/dL)		5.7 ± 0.4	5.7 ± 0.4	5.7 ± 0.3	5.8 ± 0.3	5.8 ± 0.3
TG (mg/dL)		130 ± 12	131 ± 16	131 ± 13	129 ± 12	129 ± 10
CK (U/L)		252 ± 48	269 ± 46	259 ± 47	269 ± 49	269 ± 46

Data are expressed as mean ± SD (*n* = 10 mice per group). AST, aspartate aminotransferase; ALT, alanine transaminase; ALB, albumin; BUN, blood urea nitrogen; CREA, creatinine; UA, uric acid; TP, total protein; TG, triacylglycerol; CK, creatine kinase.

**Table 3:** Effect of SBE supplementation on body weight, body composition, and water and diet intake.

Characteristics	Vehicle	Isocaloric	SBE-1X	SBE-2X	SBE-4X
Initial BW (g)	29.9 ± 0.7	29.7 ± 0.6	29.7 ± 0.9	29.7 ± 0.7	29.7 ± 0.4
1st wk BW	33.8 ± 1.1	33.5 ± 1.4	33.3 ± 0.7	33.4 ± 1.3	33.4 ± 1.2
2nd wk BW	35.5 ± 1.9	35.5 ± 1.4	35.2 ± 1.4	34.8 ± 1.6	34.4 ± 1.3
3rd wk BW	36.6 ± 2.0	36.8 ± 2.0	36.7 ± 2.0	36.2 ± 2.2	35.7 ± 1.5
4th wk BW	37.4 ± 2.4	37.6 ± 2.1	37.5 ± 2.2	36.9 ± 2.3	36.5 ± 1.7
5th wk BW	37.9 ± 2.5	38.4 ± 2.1	37.9 ± 2.3	37.4 ± 2.2	36.9 ± 1.7
Final BW (g)	38.8 ± 2.7	39.0 ± 2.2	39.0 ± 1.6	38.5 ± 2.2	38.0 ± 1.2
Water intake (mL/mouse/day)	7.1 ± 0.4	7.2 ± 0.4	7.2 ± 0.5	7.1 ± 0.6	7.2 ± 0.5
Diet (g/mouse/day)	6.1 ± 0.9	6.2 ± 0.9	6.3 ± 0.8	6.1 ± 0.9	6.3 ± 0.7
Calorie intake from diet (Chow 5001) (Kcal/mouse/day) (A)	20.5 ± 3.1	20.8 ± 2.9	21.2 ± 2.8	20.4 ± 3.0	21.1 ± 2.4
Calorie intake from supplements (Kcal/mouse/day) (B)	0.0 ± 0.0 ^a^	0.1 ± 0.0 ^b^	0.1 ± 0.0 ^b^	0.3 ± 0.0 ^c^	0.5 ± 0.1 ^c^
Total daily calorie intake (Kcal/mouse/day) (A) + (B)	20.5 ± 3.1	20.9 ± 2.9	21.3 ± 2.8	20.7 ± 3.0	21.7 ± 2.4
Liver (g)	2.34 ± 0.30	2.31 ± 0.30	2.25 ± 0.21	2.29 ± 0.31	2.35 ± 0.16
Kidney (g)	0.64 ± 0.06	0.64 ± 0.08	0.64 ± 0.05	0.63 ± 0.05	0.63 ± 0.04
Muscle (g)	0.37 ± 0.03	0.38 ± 0.02	0.39 ± 0.04	0.36 ± 0.05	0.36 ± 0.03
Heart (g)	0.21 ± 0.03	0.21 ± 0.02	0.23 ± 0.02	0.21 ± 0.02	0.21 ± 0.02
Lung (g)	0.26 ± 0.03	0.26 ± 0.03	0.26 ± 0.03	0.26 ± 0.03	0.26 ± 0.04
EFP (g)	0.44 ± 0.08	0.43 ± 0.07	0.44 ± 0.05	0.43 ± 0.07	0.43 ± 0.05
BAT (g)	0.11 ± 0.03	0.10 ± 0.02	0.11 ± 0.02	0.11 ± 0.02	0.09 ± 0.02
Relative liver weight (%)	5.98 ± 0.38	5.86 ± 0.54	5.73 ± 0.57	5.88 ± 0.59	6.14 ± 0.27
Relative kidney weight (%)	1.63 ± 0.20	1.63 ± 0.17	1.62 ± 0.07	1.64 ± 0.14	1.64 ± 0.10
Relative muscle weight (%)	0.96 ± 0.10	0.98 ± 0.05	0.98 ± 0.12	0.94 ± 0.12	0.95 ± 0.08
Relative heart weight (%)	0.55 ± 0.07	0.52 ± 0.06	0.58 ± 0.07	0.54 ± 0.05	0.54 ± 0.05
Relative lung weight (%)	0.67 ± 0.08	0.67 ± 0.09	0.66 ± 0.07	0.66 ± 0.05	0.68 ± 0.11
Relative EFP weight (%)	1.12 ± 0.18	1.10 ± 0.17	1.11 ± 0.12	0.95 ± 0.03	0.93 ± 0.08
Relative BAT weight (%)	0.28 ± 0.07	0.26 ± 0.05	0.27 ± 0.04	0.28 ± 0.06	0.24 ± 0.05

Data are expressed as mean  ±  SD (*n*  =  10 mice per group). EFP, epididymal fat pad; BAT, brown adipose tissue. The different superscript letters (a, b, c) in the same row represent significant difference at *p* < 0.05.

**Table 4:** Nutritional content of the SBE supplement.

Nutrition Facts	100 mL SBE
Total calories (kcal)	30.4
Protein (g/100 mL)	7.6
Fat (g/100 mL)	-
Saturated fat (g/100 mL)	-
Trans fat (g/100 mL)	-
Moisture (g/100 mL)	94.0
Sodium (mg/100 mL)	39.6
Carbohydrate (g/100 mL)	-
**Total BCAA** (leucine, isoleucine, and valine)	6.86% in protein

## Data Availability

The data presented in this study are available within the article.

## References

[1] Wan J.J., Qin Z., Wang P.Y., Sun Y., Liu X. (2017). Muscle fatigue: General understanding and treatment. Exp. Mol. Med..

[2] Vashistha V., Singh B., Kaur S., Prokop L.J., Kaushik D. (2016). The Effects of Exercise on Fatigue, Quality of Life, and Psychological Function for Men with Prostate Cancer: Systematic Review and Meta-analyses. Eur. Urol. Focus..

[3] Gonzalez J.T., Fuchs C.J., Betts J.A., van Loon L.J. (2016). Liver glycogen metabolism during and after prolonged endurance-type exercise. Am. J. Physiol. Endocrinol. Metab..

[4] Powers S.K., Nelson W.B., Hudson M.B. (2011). Exercise-induced oxidative stress in humans: Cause and consequences. Free Radic. Biol. Med..

[5] Chen Y.J., Baskaran R., Shibu M.A., Lin W.T. (2022). Anti-Fatigue and Exercise Performance Improvement Effect of *Glossogyne tenuifolia* Extract in Mice. Nutrients.

[6] Schwartz A.L. (1999). Fatigue mediates the effects of exercise on quality of life. Qual. Life Res..

[7] Aoi W., Naito Y., Yoshikawa T. (2006). Exercise and functional foods. Nutr. J..

[8] Chen Y., Michalak M., Agellon L.B. (2018). Importance of Nutrients and Nutrient Metabolism on Human Health. Yale J. Biol. Med..

[9] Guo Z., Lin D., Guo J., Zhang Y., Zheng B. (2017). In Vitro Antioxidant Activity and In Vivo Anti-Fatigue Effect of Sea Horse (*Hippocampus*) Peptides. Molecules.

[10] Huang S., Lin H., Deng S.G. (2015). Study of Anti-Fatigue Effect in Rats of Ferrous Chelates Including Hairtail Protein Hydrolysates. Nutrients.

[11] Borow K.M., Nelson J.R., Mason R.P. (2015). Biologic plausibility, cellular effects, and molecular mechanisms of eicosapentaenoic acid (EPA) in atherosclerosis. Atherosclerosis.

[12] Tacon A.G., Metian M. (2018). Food matters: Fish, income, and food supply—A comparative analysis. Rev. Fish. Sci. Aquac..

[13] Gajanan P.G., Elavarasan K., Shamasundar B.A. (2016). Bioactive and functional properties of protein hydrolysates from fish frame processing waste using plant proteases. Environ. Sci. Pollut. Res. Int..

[14] Phadke G.G., Rathod N.B., Ozogul F., Elavarasan K., Karthikeyan M., Shin K.H., Kim S.K. (2021). Exploiting of Secondary Raw Materials from Fish Processing Industry as a Source of Bioactive Peptide-Rich Protein Hydrolysates. Mar. Drugs.

[15] Manninen A.H. (2004). Protein hydrolysates in sports and exercise: A brief review. J. Sports Sci. Med..

[16] Majidiyan N., Hadidi M., Azadikhah D., Moreno A. (2022). Protein complex nanoparticles reinforced with industrial hemp essential oil: Characterization and application for shelf-life extension of Rainbow trout fillets. Food Chem. X.

[17] Bertrais S., Galan P., Renault N., Zarebska M., Preziosi P., Hercberg S. (2001). Consumption of soup and nutritional intake in French adults: Consequences for nutritional status. J. Hum. Nutr. Diet..

[18] Leeb E., Kulozik U., Cheison S. (2011). Thermal pre-treatment of β-Lactoglobulin as a tool to steer enzymatic hydrolysis and control the release of peptides. Procedia Food Sci..

[19] Lin L., Tao N., Su H., Zhang J., Zhong J. (2020). Migration of nutrients and formation of micro/nano-sized particles in Atlantic salmon (*Salmo salar*) and bighead carp (*Aristichthys nobilis*) head soups. Food Biosci..

[20] Rengpipat S., Rueangruklikhit T., Piyatiratitivorakul S. (2008). Evaluations of lactic acid bacteria as probiotics for juvenile seabass *Lates calcarifer*. Aquac. Res..

[21] Munekata P.E.S., Pateiro M., Domínguez R., Zhou J., Barba F.J., Lorenzo J.M. (2020). Nutritional Characterization of Sea Bass Processing By-Products. Biomolecules.

[22] Lekjing S., Venkatachalam K., Wangbenmad C. (2021). Biochemical evaluation of novel seabass (*Lates calcarifer*) fish essence soup prepared by prolonged boiling process. Arabian J. Chem..

[23] Hsu T.H., Chiu C.C., Wang Y.C., Chen T.H., Chen Y.H., Lee Y.P., Hung S.W., Wu C.P., Chuang H.L. (2018). Supplementation with Beef Extract Improves Exercise Performance and Reduces Post-Exercise Fatigue Independent of Gut Microbiota. Nutrients.

[24] Huang S.W., Hsu Y.J., Lee M.C., Li H.S., Yeo P.C.W., Lim A.L., Huang C.C. (2018). In Vitro and In Vivo Functional Characterization of Essence of Chicken as an Ergogenic Aid. Nutrients.

[25] Ryan J.T., Ross R.P., Bolton D., Fitzgerald G.F., Stanton C. (2011). Bioactive peptides from muscle sources: Meat and fish. Nutrients.

[26] Samples S. (2014). Towards a more sustainable production of fish as an important source for human nutrition. J. Fish. Livest. Prod..

[27] Li P., Mai K., Trushenski J., Wu G. (2009). New developments in fish amino acid nutrition: Towards functional and environmentally oriented aquafeeds. Amino Acids.

[28] Manoli I., Venditti C.P. (2016). Disorders of branched chain amino acid metabolism. Transl. Sci. Rare Dis..

[29] Adeva-Andany M.M., López-Maside L., Donapetry-García C., Fernández-Fernández C., Sixto-Leal C. (2017). Enzymes involved in branched-chain amino acid metabolism in humans. Amino Acids.

[30] Felig P., Wahren J. (1971). Amino acid metabolism in exercising man. J. Clin. Investig..

[31] Ahlborg G., Felig P., Hagenfeldt L., Hendler R., Wahren J. (1974). Substrate turnover during prolonged exercise in man. Splanchnic and leg metabolism of glucose, free fatty acids, and amino acids. J. Clin. Investig..

[32] De Campos-Ferraz P.L., Ribeiro S.M., Luz Sdos S., Lancha A.H., Tirapegui J. (2011). Exercise x BCAA Supplementation in Young Trained Rats: What are their Effects on Body Growth?. J. Sports Sci. Med..

[33] De Araujo J.A., Falavigna G., Rogero M.M., Pires I.S., Pedrosa R.G., Castro I.A., Donato J., Tirapegui J. (2006). Effect of chronic supplementation with branched-chain amino acids on the performance and hepatic and muscle glycogen content in trained rats. Life Sci..

[34] Cantó C., Auwerx J. (2009). PGC-1alpha, SIRT1 and AMPK, an energy sensing network that controls energy expenditure. Curr. Opin. Lipidol..

[35] Marcinko K., Steinberg G.R. (2014). The role of AMPK in controlling metabolism and mitochondrial biogenesis during exercise. Exp. Physiol..

[36] Gibala M.J., Little J.P., Macdonald M.J., Hawley J.A. (2012). Physiological adaptations to low-volume, high-intensity interval training in health and disease. J. Physiol..

[37] Ørtenblad N., Westerblad H., Nielsen J. (2013). Muscle glycogen stores and fatigue. J. Physiol..

[38] Shimomura Y., Murakami T., Nakai N., Nagasaki M., Obayashi M., Li Z., Xu M., Sato Y., Kato T., Shimomura N. (2000). Supression of glycogen consumption during acute exercise by dietary branched-chain amino acids in rats. J. Nutr. Sci. Vitamol..

[39] Matsumoto K., Koba T., Hamada K., Tsujimoto H., Mitsuzono R. (2009). Branched-chain amino acid supplementation increases the lactate threshold during an incremental exercise test in trained individuals. J. Nutr. Sci. Vitaminol..

[40] Huang W.C., Lin C.I., Chiu C.C., Lin Y.T., Huang W.K., Huang H.Y., Huang C.C. (2014). Chicken essence improves exercise performance and ameliorates physical fatigue. Nutrients.

[41] Falavigna G., Alves de Araújo J., Rogero M.M., Pires I.S., Pedrosa R.G., Martins E., Alves de Castro I., Tirapegui J. (2012). Effects of diets supplemented with branched-chain amino acids on the performance and fatigue mechanisms of rats submitted to prolonged physical exercise. Nutrients.

[42] Williams M. (2005). Dietary supplements and sports performance: Amino acids. J. Int. Soc. Sports Nutr..

[43] Lin C.L., Lee M.C., Hsu Y.J., Huang W.C., Huang C.C., Huang S.W. (2018). Isolated Soy Protein Supplementation and Exercise Improve Fatigue-Related Biomarker Levels and Bone Strength in Ovariectomized Mice. Nutrients.

[44] Kim D.H., Kim S.H., Jeong W.S., Lee H.Y. (2013). Effect of BCAA intake during endurance exercises on fatigue substances, muscle damage substances, and energy metabolism substances. J. Exerc. Nutrition Biochem..

[45] Cairns S.P. (2006). Lactic acid and exercise performance: Culprit or friend?. Sports Med..

[46] De Palo E.F., Gatti R., Cappellin E., Schiraldi C., De Palo C.B., Spinella P. (2001). Plasma lactate, GH and GH-binding protein levels in exercise following BCAA supplementation in athletes. Amino Acids..

[47] MacLean D.A., Graham T.E., Saltin B. (1996). Stimulation of muscle ammonia production during exercise following branched-chain amino acid supplementation in humans. J Physiol..

[48] Holecek M., Kandar R., Sispera L., Koverik M. (2011). Acute hyperammonemia activates branched-chain amino acid catabolism and decreases their extracellular concentrations: Different sensitivity of red and white muscle. Amino Acids.

[49] Kitaoka Y. (2014). McArdle Disease and Exercise Physiology. Biology.

[50] Korzeniewski B. (2006). AMP deamination delays muscle acidification during heavy exercise and hypoxia. J. Biol. Chem..

[51] Mikulski T., Dabrowski J., Hilgier W., Ziemba A., Krzeminski K. (2015). Effects of supplementation with branched chain amino acids and ornithine aspartate on plasma ammonia and central fatigue during exercise in healthy men. Folia Neuropathol..

[52] Kohno K., Hayakawa F., Xichang W., Shunsheng C., Yokoyama M., Kasai M., Takeutchi F., Hatae K. (2005). Comparative study on flavor preference between Japanese and Chinese for dried bonito stock and chicken bouillon. J. Food Sci..

[53] Hsu Y.J., Lee M.C., Huang C.C., Ho C.S. (2021). The effects of different types of aquatic exercise training interventions on a high-fructose diet-fed mice. Int. J. Med. Sci..

[54] Hsu Y.J., Jhang W.L., Lee M.C., Bat-Otgon B., Narantungalag E., Huang C.C. (2021). Lactose-riched Mongolian mare’s milk improves physical fatigue and exercise performance in mice. Int. J. Med. Sci..

[55] Lee M.C., Hsu Y.J., Ho H.H., Kuo Y.W., Lin W.Y., Tsai S.Y., Chen W.L., Lin C.L., Huang C.C. (2021). Effectiveness of human-origin *Lactobacillus plantarum* PL-02 in improving muscle mass, exercise performance and anti-fatigue. Sci. Rep..

