# Correction: Tai et al. Sea Bass Essence from *Lates calcarifer* Improves Exercise Performance and Anti-Fatigue in Mice. *Metabolites* 2022, *12*, 531

**DOI:** 10.3390/metabo15010046

**Published:** 2025-01-13

**Authors:** Hong-Jun Tai, Mon-Chien Lee, Yi-Ju Hsu, Chun-Yen Kuo, Chi-Chang Huang, Ming-Fu Wang

**Affiliations:** 1 Department of Food and Nutrition, Providence University, Taichung 43301, Taiwan; yeast2136@gmail.com; 2 Graduate Institute of Sports Science, National Taiwan Sport University, Taoyuan City 33301, Taiwan; 1061304@ntsu.edu.tw (M.-C.L.); ruby780202@ntsu.edu.tw (Y.-J.H.); 3 Program in Health and Social Welfare for Indigenous Peoples, Providence University, Taichung 43301, Taiwan; cykuo2@pu.edu.tw

## 1. Error in Figure/Table

First, in the original publication [1], there was a mistake in Table 1 as published. The data in Table 1 were wrong and the group name was wrongly marked; SBE-5X should be changed to SBE-4X. The corrected Table 1 appears below.

Second, in the original publication, there was a mistake in Table 2 as published. There was a unit error for ALB and TP and the group name was wrongly marked; SBE-5X should be changed to SBE-4X. The corrected Table 2 appears below. 

Third, in the original publication, there was a mistake in Table 3 as published. The group name was wrongly marked; SBE-5X should be changed to SBE-4X. The corrected Table 3 appears below.

Fourth, in the original publication, there was a mistake in Figure 4 as published. The group name was wrongly marked, SBE-5X should be changed to SBE-4X. The corrected Figure 4 appears below.

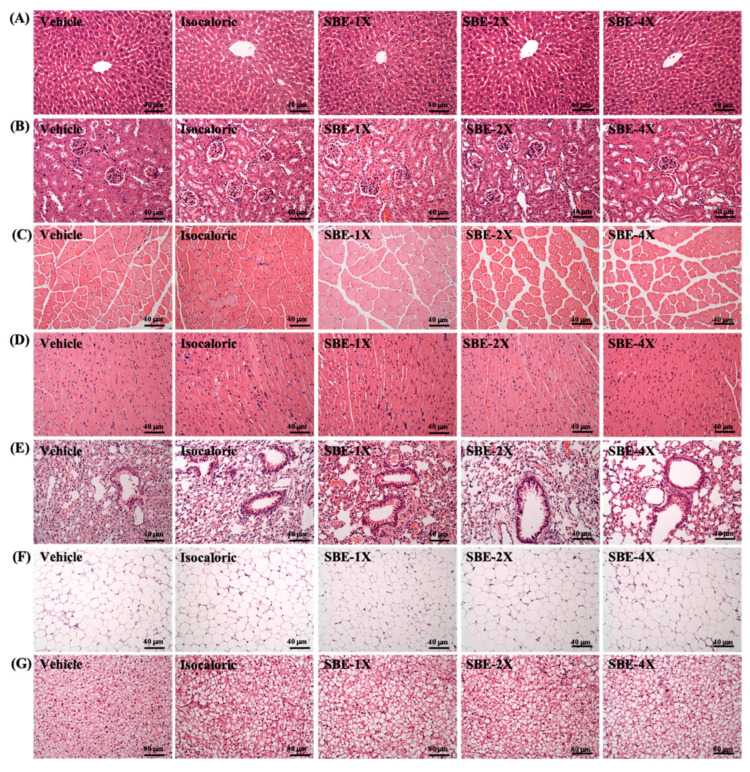



## 2. Text Correction

There was an error in the original publication. The group name was wrongly marked; SBE-5X should be changed to SBE-4X.

A correction has been made to Section 2; Results Section 2.3. Effect of SBE Supplementation on Fatigue-Related Biochemical Indicators after the 10-min Swim Test or a 90-min Swim Test and a 60-min Rest, Paragraphs 1 and 2:

We also evaluated the NH_3_ and BUN concentration after the 10-min swim test. As shown in Figure 2A, the NH_3_ levels in the vehicle, isocaloric, SBE-1X, SBE-2X, and SBE-4X groups were 167 ± 18, 144 ± 19, 145 ± 17, 133 ± 18, and 148 ± 17 (umol/L), respectively. The SBE-1X, SBE-2X, and SBE-4X groups were significantly lower than the vehicle group, i.e., by 13.11% (*p* = 0.0089), 20.42% (*p* = 0.0001), and 11.38% (*p* = 0.0221), respectively, but no dose-dependent trend was observed.

We measured the serum BUN level after the 90-min swim test followed by 60 min of rest. As shown in Figure 2B, the BUN levels in the SBE-1X, SBE-2X, and SBE-4X groups were 46.5 ± 1.8, 46.5 ± 2.0, 38.5 ± 2.2, 35.5 ± 2.2, and 34.8 ± 1.5 (mg/dL), respectively. Compared with the vehicle group, the SBE-1X, SBE-2X, and SBE-4X groups were significantly lower by 17.26% (*p* < 0.0001), 23.78% (*p* < 0.0001), and 25.22% (*p* < 0.0001), respectively. In addition, they were significantly lower than the isocaloric group by 17.27% (*p* < 0.0001), 23.79% (*p* < 0.0001), and 25.23% (*p* < 0.0001), respectively. For the trend analysis, serum BUN levels after the 90-min swim test followed by 60 min of rest had decreased dose-dependently with SBE supplementation (*p* < 0.0001).

In addition, a correction has been made to Section 2; Results Section, 2.4. Effect of SBE Supplementation on Liver and Muscle Glycogen Contents, Paragraphs 1 and 2:

The liver glycogen content in the vehicle, isocaloric, SBE-1X, SBE-2X, and SBE-4X groups were 15.87 ± 0.93, 15.69 ± 1.24, 20.76 ± 3.65, 23.24 ± 1.98, and 23.97 ± 0.51 (mg/g liver), respectively. The SBE-1X, SBE-2X, and SBE-4X groups were significantly greater than the vehicle group by 1.31-fold (*p* < 0.0001), 1.46-fold (*p* < 0.0001) and 1.51-fold (*p* < 0.0001), respectively, and also were significantly greater than the isocaloric group by 1.32-fold (*p* < 0.0001), 1.48-fold (*p* < 0.0001) and 1.53-fold (*p* < 0.0001), respectively (Figure 3A).

The muscle glycogen content in the vehicle, isocaloric, SBE-1X, SBE-2X, and SBE-4X groups were 1.35 ± 0.08, 1.36 ± 0.07, 1.57 ± 0.08, 1.67 ± 0.05, and 1.73 ± 0.06 (mg/g muscle), respectively. The SBE-1X, SBE-2X, and SBE-4X groups were significantly greater than the vehicle group by 1.16-fold (*p* < 0.0001), 1.24-fold (*p* < 0.0001) and 1.28-fold (*p* < 0.0001), respectively, and were also significantly greater than the isocaloric group by 1.15-fold (*p* < 0.0001), 1.22-fold (*p* < 0.0001) and 1.26-fold (*p* < 0.0001), respectively (Figure 3A).

The authors state that the scientific conclusions are unaffected. This correction was approved by the Academic Editor. The original publication has also been updated.

## Figures and Tables

**Table 1:** Effects of SBE supplementation on serum levels of lactate after the 10 min swim test. The lactate production rate (B/A) was the value of the lactate level after exercise (B) divided by that before exercise (A). The clearance rate (B − C)/B was defined as the lactate level after swimming (B) minus that after 20 min of rest (C) divided by that after swimming (B). Data are expressed as mean ± SD (*n* = 10 mice per group). Values in the same row with different superscript letters (a, b, c, d) differ significantly between groups, *p* < 0.05.

	Groups	Vehicle	Isocaloric	SBE-1X	SBE-2X	SBE-4X
Time Point		Lactate (mmol/L)				
Before swimming (A)		3.81 ± 0.42 ^a^	3.69 ± 0.46 ^a^	3.70 ± 0.35 ^a^	3.77 ± 0.49 ^a^	3.86 ± 0.51 ^a^
After swimming (B)		6.56 ± 0.93 ^b^	6.51 ± 1.01 ^b^	5.32 ± 0.89 ^a^	5.01 ± 0.28 ^a^	4.66 ± 0.37 ^a^
After a 20 min resting (C)		5.21 ± 0.53 ^d^	5.25 ± 0.72 ^d^	4.36 ± 0.48 ^c^	3.86 ± 0.46 ^b^	3.41 ± 0.26 ^a^
Rates of lactate production and clearance						
Production rate = B/A		1.73 ± 0.25 ^c^	1.77 ± 0.21 ^c^	1.45 ± 0.28 ^b^	1.34 ± 0.14 ^ab^	1.22 ± 0.11 ^a^
Clearance rate = (B − C)/B		0.19 ± 0.12 ^a^	0.18 ± 0.13 ^a^	0.16 ± 0.16 ^a^	0.23 ± 0.09 ^a^	0.26 ± 0.08 ^a^

**Table 2:** Effects of SBE supplementation on biochemical parameters.

	Groups	Vehicle	Isocaloric	SBE-1X	SBE-2X	SBE-4X
Parameters						
AST (U/L)		73 ± 11	71 ± 7	75 ± 8	72 ± 6	74 ± 5
ALT (U/L)		47 ± 5	47 ± 5	49 ± 5	47 ± 5	47 ± 5
ALB (g/dL)		3.44 ± 0.11	3.28 ± 0.23	3.37 ± 0.24	3.37 ± 0.32	3.35 ± 0.21
BUN (mg/dL)		27.1 ± 3.7	26.1 ± 1.9	26.3 ± 2.3	26.2 ± 2.6	26.3 ± 2.7
CREA (mg/dL)		0.43 ± 0.02	0.43 ± 0.02	0.44 ± 0.03	0.44 ± 0.03	0.43 ± 0.03
UA (mg/dL)		2.1 ± 0.8	2.1 ± 0.5	2.0 ± 0.5	2.2 ± 0.4	2.1 ± 0.8
TP (g/dL)		5.7 ± 0.4	5.7 ± 0.4	5.7 ± 0.3	5.8 ± 0.3	5.8 ± 0.3
TG (mg/dL)		130 ± 12	131 ± 16	131 ± 13	129 ± 12	129 ± 10
CK (U/L)		252 ± 48	269 ± 46	259 ± 47	269 ± 49	269 ± 46

Data are expressed as mean ± SD (*n* = 10 mice per group). AST, aspartate aminotransferase; ALT, alanine transaminase; ALB, albumin; BUN, blood urea nitrogen; CREA, creatinine; UA, uric acid; TP, total protein; TG, triacylglycerol; CK, creatine kinase.

**Table 3:** Effect of SBE supplementation on body weight, body composition, and water and diet intake.

Characteristics	Vehicle	Isocaloric	SBE-1X	SBE-2X	SBE-4X
Initial BW (g)	29.9 ± 0.7	29.7 ± 0.6	29.7 ± 0.9	29.7 ± 0.7	29.7 ± 0.4
1st wk BW	33.8 ± 1.1	33.5 ± 1.4	33.3 ± 0.7	33.4 ± 1.3	33.4 ± 1.2
2nd wk BW	35.5 ± 1.9	35.5 ± 1.4	35.2 ± 1.4	34.8 ± 1.6	34.4 ± 1.3
3rd wk BW	36.6 ± 2.0	36.8 ± 2.0	36.7 ± 2.0	36.2 ± 2.2	35.7 ± 1.5
4th wk BW	37.4 ± 2.4	37.6 ± 2.1	37.5 ± 2.2	36.9 ± 2.3	36.5 ± 1.7
5th wk BW	37.9 ± 2.5	38.4 ± 2.1	37.9 ± 2.3	37.4 ± 2.2	36.9 ± 1.7
Final BW (g)	38.8 ± 2.7	39.0 ± 2.2	39.0 ± 1.6	38.5 ± 2.2	38.0 ± 1.2
Water intake (mL/mouse/day)	7.1 ± 0.4	7.2 ± 0.4	7.2 ± 0.5	7.1 ± 0.6	7.2 ± 0.5
Diet (g/mouse/day)	6.1 ± 0.9	6.2 ± 0.9	6.3 ± 0.8	6.1 ± 0.9	6.3 ± 0.7
Calorie intake from diet (Chow 5001) (Kcal/mouse/day) (A)	20.5 ± 3.1	20.8 ± 2.9	21.2 ± 2.8	20.4 ± 3.0	21.1 ± 2.4
Calorie intake from supplements (Kcal/mouse/day) (B)	0.0 ± 0.0 ^a^	0.1 ± 0.0 ^b^	0.1 ± 0.0 ^b^	0.3 ± 0.0 ^c^	0.5 ± 0.1 ^c^
Total daily calorie intake (Kcal/mouse/day) (A) + (B)	20.5 ± 3.1	20.9 ± 2.9	21.3 ± 2.8	20.7 ± 3.0	21.7 ± 2.4
Liver (g)	2.34 ± 0.30	2.31 ± 0.30	2.25 ± 0.21	2.29 ± 0.31	2.35 ± 0.16
Kidney (g)	0.64 ± 0.06	0.64 ± 0.08	0.64 ± 0.05	0.63 ± 0.05	0.63 ± 0.04
Muscle (g)	0.37 ± 0.03	0.38 ± 0.02	0.39 ± 0.04	0.36 ± 0.05	0.36 ± 0.03
Heart (g)	0.21 ± 0.03	0.21 ± 0.02	0.23 ± 0.02	0.21 ± 0.02	0.21 ± 0.02
Lung (g)	0.26 ± 0.03	0.26 ± 0.03	0.26 ± 0.03	0.26 ± 0.03	0.26 ± 0.04
EFP (g)	0.44 ± 0.08	0.43 ± 0.07	0.44 ± 0.05	0.43 ± 0.07	0.43 ± 0.05
BAT (g)	0.11 ± 0.03	0.10 ± 0.02	0.11 ± 0.02	0.11 ± 0.02	0.09 ± 0.02
Relative liver weight (%)	5.98 ± 0.38	5.86 ± 0.54	5.73 ± 0.57	5.88 ± 0.59	6.14 ± 0.27
Relative kidney weight (%)	1.63 ± 0.20	1.63 ± 0.17	1.62 ± 0.07	1.64 ± 0.14	1.64 ± 0.10
Relative muscle weight (%)	0.96 ± 0.10	0.98 ± 0.05	0.98 ± 0.12	0.94 ± 0.12	0.95 ± 0.08
Relative heart weight (%)	0.55 ± 0.07	0.52 ± 0.06	0.58 ± 0.07	0.54 ± 0.05	0.54 ± 0.05
Relative lung weight (%)	0.67 ± 0.08	0.67 ± 0.09	0.66 ± 0.07	0.66 ± 0.05	0.68 ± 0.11
Relative EFP weight (%)	1.12 ± 0.18	1.10 ± 0.17	1.11 ± 0.12	0.95 ± 0.03	0.93 ± 0.08
Relative BAT weight (%)	0.28 ± 0.07	0.26 ± 0.05	0.27 ± 0.04	0.28 ± 0.06	0.24 ± 0.05

Data are expressed as mean  ±  SD (*n*  =  10 mice per group). EFP, epididymal fat pad; BAT, brown adipose tissue. The different superscript letters (a, b, c) in the same row represent significant difference at *p* < 0.05.
